# A gene expression atlas for different kinds of stress in the mouse brain

**DOI:** 10.1038/s41597-020-00772-z

**Published:** 2020-12-16

**Authors:** Tiziano Flati, Silvia Gioiosa, Giovanni Chillemi, Andrea Mele, Alberto Oliverio, Cecilia Mannironi, Arianna Rinaldi, Tiziana Castrignanò

**Affiliations:** 1 CINECA, SuperComputing Applications and Innovation Department, Via dei Tizii 6, 00185 Roma, Italy; 2 DIBAF, University of Tuscia, 01100 Viterbo, Italy; 3 Institute of Biomembranes, Bioenergetics and Molecular Biotechnologies, IBIOM, CNR, Bari, Italy; 4 Department of Biology and Biotechnology “C. Darwin”, Sapienza University of Rome, Rome, Italy; 5 Center for Research in Neurobiology “D. Bovet”, Sapienza University of Rome, Rome, Italy; 6 Istituto di Biologia e Patologia Molecolari, CNR, Rome, Italy; 7 Department of Ecological and Biological Sciences (DEB), University of Tuscia, Largo Università snc, 01100 Viterbo, Italy

**Keywords:** Molecular neuroscience, Gene ontology, Data processing, Transcriptomics

## Abstract

Stressful experiences are part of everyday life and animals have evolved physiological and behavioral responses aimed at coping with stress and maintaining homeostasis. However, repeated or intense stress can induce maladaptive reactions leading to behavioral disorders. Adaptations in the brain, mediated by changes in gene expression, have a crucial role in the stress response. Recent years have seen a tremendous increase in studies on the transcriptional effects of stress. The input raw data are freely available from public repositories and represent a wealth of information for further global and integrative retrospective analyses. We downloaded from the Sequence Read Archive 751 samples (SRA-experiments), from 18 independent BioProjects studying the effects of different stressors on the brain transcriptome in mice. We performed a massive bioinformatics re-analysis applying a single, standardized pipeline for computing differential gene expression. This data mining allowed the identification of novel candidate stress-related genes and specific signatures associated with different stress conditions. The large amount of computational results produced was systematized in the interactive “Stress Mice Portal”.

## Introduction

Exposure to aversive or potentially life-threatening events, elicits a coordinated and evolutionary conserved series of physiological and behavioural responses aimed at coping with the stressful experience and re-establishing homeostasis^1,2^. However, under certain circumstances, stress induces maladaptive responses in susceptible individuals that lead to long-lasting mental disorders, such as depression, anxiety or post-traumatic stress disorder (PTSD), turning into elevated costs for the health system worldwide^1^.

The brain, together with the immune, metabolic and neuroendocrine systems, is a major player in the response to stressful experiences^3–6^. Stress activates the sympathetic division of the autonomic nervous system, inducing the release of noradrenaline and adrenaline from the adrenal medulla and of noradrenaline from the sympathetic nerves^5^. At the same time, stress acts on the hypothalamic-pituitary-adrenal (HPA) axis, culminating in the release of circulating corticosteroids from the adrenal cortex. The quick increase in catecholamines and corticosteroids concentration triggers a cascade of responses that affect the whole body and allow to perceive and react to the stressors. Corticosteroids, by binding to mineralocorticoid and glucocorticoid receptors widely expressed in the brain, induce also slower but more durable changes in gene expression, that lead to functional and structural alterations in cortical, limbic and midbrain regions^3,6^.

It is increasingly clear that neural adaptations following a stressful experience depend on the specific characteristics of the stressor and the subject, as well as on the brain region involved, the stage of life, previous exposure to stress and individual differences in stress resilience^6–9^.

Animal models have proven instrumental in defining many aspects of the transcriptional response of the brain to stress, largely because of the inherent limitations posed by direct brain examination in humans and the scarcity of post mortem human brain tissue banks. In rodents stress can be induced by chronic social defeat, exposure to a predator or a predator’s scent, exposure to an intruder, social isolation, housing instability, maternal separation or restraint. Other stressors, such as immobilization, electric foot-shock, exposure to cold water or a cold environment, and forced swimming, present a stronger physical component. These models can also reproduce many other attributes of stress, such as frequency, duration, intensity, associated pain, predictability or escapability and allow the quantification of a range of negative behavioral outcomes such as social withdrawal, fear, anxiety-like behavior, anhedonia and thus are often used as preclinical models of depression or PTSD^7,10,11^.

Different experimental stress conditions seem to regulate largely different sets of genes in the rodent brain^6,12–16^. For example, Gray and collaborators^13^ reported a minimal overlap in the gene expression profiles associated with acute forced swimming stress, chronic restraint or a combination of the two, in the hippocampus (HIPP). Highly distinct gene expression patterns were observed between the dorsal and the ventral HIPP after different kinds of acute stress^16^ or in the nucleus accumbens (NACC) of male and female subjects after subchronic variable stress^15^.

Methodological differences in sample preparation, RNA expression quantification and data analysis among independent studies, further complicate the accurate definition of the main pathways involved in stress reactivity^17^.

With the aim of identifying convergent biological processes and signalling pathways affected by stress and revealing possible novel candidate genes, we set out to analyze the transcriptional profile associated with several stress conditions. In recent years there has been a shift in the approach adopted for the characterization of the transcriptome from microarray to deep sequencing, which on the one hand provides a far more precise and extensive characterization of the level of RNA transcripts as well as the discovery of novel transcripts, but on the other hand poses substantial computational challenges. As reported by Rung *et al*. in 2012^18^, public gene expression data are commonly reused to address biological questions by reanalyzing primary data. Approximately half of these studies use public gene expression data without adding newly generated data. Reuse of RNA-sequencing (RNA-seq) data allows to reduce the costs of the biological experiments and get further information from the quantitative nature of sequencing-based expression data. Moreover, this approach has the additional benefit of helping reduce the number of animals used in biomedical research^19^. Therefore, we surveyed published RNA-seq transcriptomic studies obtained from the brain of mice exposed to different kinds of stress protocols.

The raw sequencing data were collected from the Sequence Read Archive (SRA) of the National Center for Biotechnology Information (NCBI). We selected 751 samples (named SRA-experiments in SRA) (332 Paired End and 459 Single End) belonging to 18 different BioProjects, for a total of 2.5 TB in size.

Since each of the laboratories producing these 18 datasets analyzed their own data by following different protocols (e.g. in terms of adopted workflows, software, genomic or gene annotation versions, thresholds of significance), we decided to re-analyze the data by applying the same transcriptomic computational workflow to the whole dataset in order to obtain a more comparable set of results. The project required a rich bioinformatics software environment and a High Performance Computing (HPC) infrastructure to download, store and re-analyze input data.

The large amount of computational data produced have been systematized in an open-source web portal available at the following link: http://hpc-bioinformatics.cineca.it/stress_mice/.

## Results

### Analysis of stress-related changes in the brain transcriptome

#### Dataset description

Briefly, we queried SRA using several stress- and brain-related keywords, then we further refined our selection based on *a priori* criteria, as described in Material and Methods and shown on the Stress Mouse Portal (http://hpc-bioinformatics.cineca.it/stress_mice/dataset_phenotypic). This process resulted in the selection of 18 BioProjects, for a total of 751 SRA-experiments (Table 1).

**Table 1:** Dataset collection.

Bioproject	Reference	Layout	#SRA-Experiments	Size (GB)	Platform (Illumina HiSeq)
PRJNA267703 ^77^	Peixoto *et al*.^34^	PE	15	130.2	2000
PRJNA281134^78^	Kao *et al*.^35^	SE	36	25.4	2000
PRJNA292861^79^	Cho *et al*.^33^	SE	15	140.0	2000
PRJNA293822^80^	Bagot *et al*.^26^	SE	140	515.1	2000
PRJNA309704^81^	Li *et al*.^20^	SE	6	9.6	2500
PRJNA322294^82^	Bagot *et al*.^27^	PE	47	107.2	2500
PRJNA323485^83^	Bondar *et al*.^28^	PE	12	57.1	2000
PRJNA336339^84^	Hodes *et al*.^15^	SE	12	30.0	2000
PRJNA352967^85^	Pena *et al*.^29^,^32^	SE,PE	144	650	2000
PRJNA391140^86^	Floriou-Servou *et al*.^16^	SE	45	75.7	4000
PRJNA398031^87^	Labonté *et al*.^24^	PE	78	320.2	2000
PRJNA401858^88^	Nasca *et al*.^22^	SE	6	10.4	2500
PRJNA415948^89^	Cheng *et al*.^23^	SE	6	6.3	2000
PRJNA430409^90^	Laine *et al*.^30^	PE,SE	87	384.1	2000
PRJNA479752^91^	Lori *et al*.^36^	SE	16	29.37	1000
PRJNA495330^92^	MacLaren *et al*.^21^	SE	24	62.27	2000
PRJNA506950^93^	Misiewicz *et al*.^31^	SE	14	39.08	500
PRJNA516641^94^	Nollet *et al*.^25^	PE	48	170.85	2500

The table shows the main characteristics of each BioProject, including the library layout and platform used for RNA-sequencing, the number of SRA-experiments selected from each BioProject and their total size (paired ends, PE; single ends, SE).

As shown in Table 2, the selected BioProjects were very heterogeneous and included several types of stress: acute^16,20^, sub-chronic^21^ or chronic^22,23^ restraint, cold swim^16^, sub-chronic^15^, or chronic^24^ variable stress, unpredictable chronic mild stress^25^, chronic social defeat^26–32^, early life stress^29,32^, electric footshock (or contextual fear conditioning)^33–35^, auditory fear conditioning with or without immobilization^36^. Fear conditioning is a classical associative memory test in mice, however it is also widely used as preclinical animal model of PTSD, as it involves delivery of mild electric footshocks that represent an aversive experience, thus it was included in our selection of stressors^37^.

**Table 2:** Dataset description.

Bioproject [#Configurations]	Type of stress^a^	Stress frequency	Time from stress	Phenotype	Brain region (Subregion)	Mouse strain	Sex	#DEGs	#Shared DEGs^b^
PRJNA309704 [1]	Restraint (30 min)	Acute	1 hr	—	HIPP	C57BL/6 J	m	48	—
PRJNA391140 [6]	*Restraint (30 min), Cold swim (6 min)*	Acute	15 min (restraint) 39 min (cold swim)	—	*HIPP* *(total, dorsal, ventral)*	C57BL/6 J	m	242	79 (33%)
PRJNA401858 [1]	Restraint (21 d, 2 hr/d)	Chronic	24 hr	—	HIPP (vDG)	C57BL/6 N	m	74	—
PRJNA415948 [1]	Restraint (15 d, 2 hr/d)	Chronic	24 hr	—	PFC	C57BL/6	m	2	—
PRJNA495330 [2]	Restraint (1 hr) plus Cold temp (2 hr), for 3 d	Subchronic	13 hr	—	*HIPP* *FC*	C57BL/6 J	m	4	0
PRJNA336339 [2]	Variable stress (mild foot shock, tail suspension and restraint, alternated over 6 d, 1 hr/d)	Subchronic	24 hr	—	NACC	C57BL/6 J	*m, f*	2	0
PRJNA398031 [4]	Variable stress (mild foot shock, tail suspension and restraint, alternated over 21 d, 1 hr/d)	Chronic	24 hr	—	*PFC* *NACC*	C57BL/6 J	*m, f*	2975	189 (6%)
PRJNA516641 [3]	Unpredictable mild stress (various mild stressorsq1, alternated daily over 9 weeks)	Chronic	15 hr	—	*HIPP* *PFC* *HT*	BALB/c	m	0	0
PRJNA293822 [24] ^c^	*Social defeat (10 d); Social defeat (10 d) followed by stress re-exposure after 28 days*	Chronic	*2 d, 28 d*	*Susceptible* *Resilient*	*HIPP (ventral)* *PFC* *NACC* *AMY*	C57BL/6 J	m	390	75 (19%)
PRJNA322294 [8]	Social defeat (10 d)	Chronic	30 d	*Susceptible* *Resilient*	*HIPP (ventral)* *PFC* *NACC* *AMY (bla)*	C57BL/6 J	m	578	32 (6%)
PRJNA323485 [2]	*Social defeat (10 or 30 d)*	Chronic	24 hr	—	PFC	C57BL/6	m	357	6 (2%)
PRJNA430409 [10] ^d^	Social defeat (10 d)	Chronic	6 d	*Susceptible* *Resilient*	*HIPP (ventral)* *PFC (m)* *BNST*	*C57BL/6Cr* *DBA/2Cr*	m	2986	872 (29%)
PRJNA506950 [2]	Social defeat (10 d)	Chronic	7 d	*Susceptible* *Resilient*	BNST	C57BL/6Cr	m	836	223
PRJNA352967 [21]	*Early life stress;* *Subthreshold variable stress (mild foot shock, tail suspension and restraint, alternated over 3 d, 1 hr/d);* *Early life stress plus Subthreshold variable stress;*	Chronic	4 d	—	*PFC* *NACC* *VTA*	C57BL/6 J	*f*	722	81 (11%)
	*Early life stress;* *Social defeat (10 d);* *Early life stress plus* *Social defeat (10 d);*	Chronic	4 d	—	*HIPP* *PFC* *NACC* *VTA*	C57BL/6 J	*m*		
PRJNA479752 [2]	*Auditory fear conditioning;* *Immobilization plus Auditory fear conditioning*	Acute	2 hr	—	AMY (bla)	C57BL/6 J	m	346	30
PRJNA267703 [2]	*Electric foot shock* *(2 s, 1.5 mA);* *Electric foot shock plus context re-exposure* *after 24 h*	Acute	30 min	—	HIPP	C57BL/6 J	m	40	17 (42%)
PRJNA281134 [6]	Electric foot shock (2 sec, 1.5 mA)	Acute	64 d	—	*HIPP (CA1) NACC* *AMY (bla,cea)* *PFC (pl,acc)*	C57BL/6 N	m	59	2 (3%)
PRJNA292861 [4]	Electric foot shock (2 sec)	Acute	*5 m, 10 m, 30 m, 4 hr*	—	HIPP	C57BL/6 N	m	17	5 (29%)

The table shows the metadata associated with each BioProject. The variables used to define the configurations for each BioProject are highlighted in italics.

(a) Each bioproject also included a non stressed control group that was used as baseline for DEGs calculations.

(b) Number of DEGs shared among at least two configurations in each bioproject.

(c) Brains from the Social defeat (10 days) plus stress re-exposure after 28 days group were dissected only at the 28 days after stress timepoint.

(d) For the strain DBA/2, there were resilient subjects only for BNST, but not for HIPP and PFC.

Brain dissection for RNA-Seq analysis was carried out at largely different intervals following stress exposure in the different BioProjects, ranging from 5 minutes to 64 days. Almost all the samples were obtained from C57BL/6 mice of either the C57BL/6 J or the C57BL/6 N substrain, one BioProject included also samples from DBA/2 mice^30^ and one from BALB/c^25^. The age of the experimental subjects ranged between 7 and 28 weeks. All studies focused on male subjects, with only three studies including groups of females^15,24,32^. Samples were obtained from the following brain regions: hippocampus (HIPP), nucleus accumbens (NACC), prefrontal cortex (PFC), amygdala (AMY), bed nucleus of stria terminalis (BNST), hypothalamus (HT) and ventral tegmental area (VTA). These brain regions are considered part of a neural circuit dysregulated in stress-related disorders, such as depression, anxiety and PTSD^6,7,38^.

**Fig. 1:**
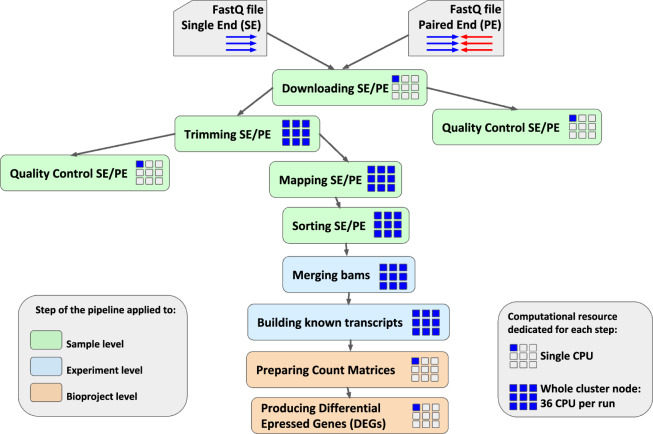
Overview of the data analysis pipeline. Raw reads were obtained in FASTQ format and were quality-assessed using FastQC program. Terminal low quality bases and adaptor sequences were trimmed off using Trimmomatic utility. Clean reads were aligned to the mouse genome (mm10*)* using HiSat2. BAM files obtained from read alignment were further processed with Stringtie in order to assemble known transcripts. Raw read counts were quantified using HTSeq. Differential expression analysis was conducted with DESeq.

Raw data from the selected BioProjects were individually re-analyzed applying the same transcriptomic pipeline (Fig. 1) and differential expression of genes was calculated by comparing samples that differed only in the exposure/non exposure to stress, but were matched with respect to all other variables, as described in detail in Material and Methods. We identified 101 unique direct comparisons (stress versus control condition) that we called configurations (Table 2). Then, in order to identify shared signatures of stress response across different conditions, we set a threshold of absolute value of log2 fold change >  = 0.38 and an adjusted p-value <  = 0.05. We reasoned that, by using a relatively low log2 fold change threshold, we could detect genes whose variation is small, but consistent in different conditions. These genes are likely to be overlooked in the analysis of individual studies, due to a bias in selecting genes with larger expression changes.

#### Gene expression analysis

**Fig. 2:**
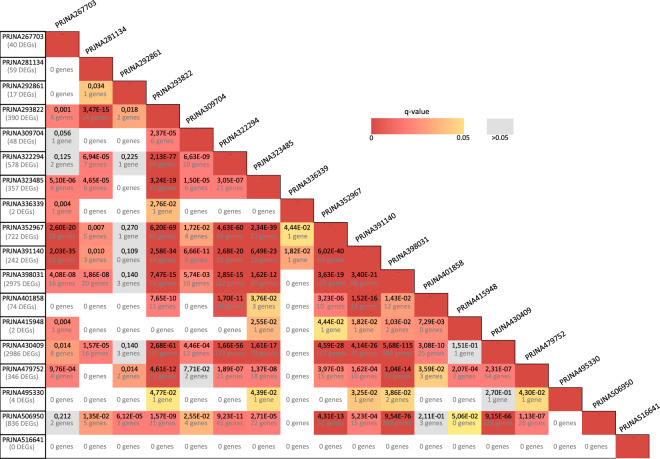
Comparison of differentially expressed genes. The table shows the significance of the intersections of DEGs between pairs of BioProjects. Cells are coloured according to the significance of the overlap (FDR corrected p-values from Fisher’s exact test lower than 0.05), with red tones indicating lower FDR corrected p-values. Grey cells correspond to non-significant overlaps (FDR corrected p-value > 0.05).

**Fig. 3:**
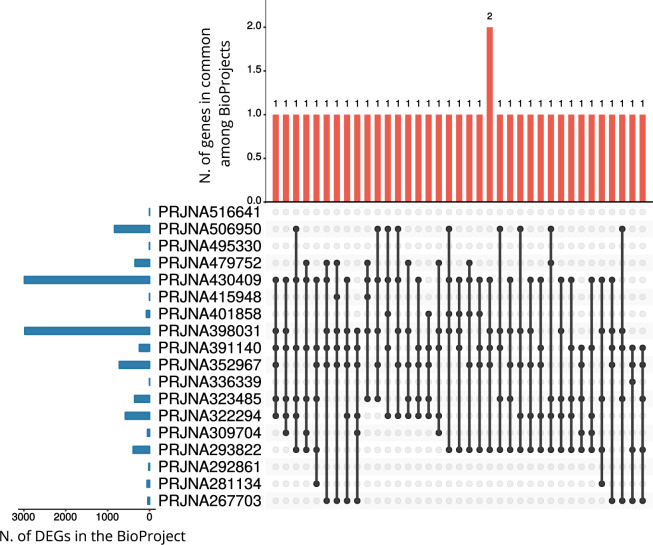
DEGs shared among BioProjects. Upset plot of the intersection of differentially expressed genes across BioProjects. Only overlaps among 6 or 5 BioProjects are shown. The horizontal bar graph on the left shows the total number of DEGs for each BioProject. The red upper bar graph shows the number of DEGs for each overlapping combination. Black connected circles indicate which BioProjects are involved in each intersection.

From the re-analysis of the 18 BioProjects we identified a total of 7,166 differentially expressed genes (DEGs), as shown in Tables 2 and 3, Supplementary Tables 1 and 2. We identified DEGs in each BioProject, except for PRJNA516641 (which is the only one containing samples from HT). The number of DEGs varied considerably among BioProjects and was not dependent on the number of configurations (Pearson’s correlation coefficient between the total number of DEGs and the number of configurations in each BioProject: R = 0.207, p = 0.41; Pearson’s correlation coefficient between the mean number of DEGs and the number of configurations in each BioProject: R = −0.091, p = 0.72). First, the lists of DEGs identified in each BioProject were compared to each other in pairs. As shown in Fig. 2, although the extent of gene overlap among most sets of DEGs was overall modest, we found several significant intersections (Fisher’s exact test, below an FDR corrected p-value threshold of 0.05), especially for the BioProjects with a higher number of DEGs (>74). Next, we performed a global analysis of DEGs intersection among all the BioProjects, with the aim of detecting genes reliably regulated in response to different stress protocols and conditions. We found 1926 genes significantly regulated in at least 2 or more BioProjects (Table 3, Supplementary Tables 1 and 2). The maximum number of BioProjects sharing at least one DEG was 6 out of 18 (Fig. 3, Table 3), while the maximum number of configurations sharing at least one DEG was 14 (out of 101 possible configurations), as shown in Table 4.

**Table 3:** DEGs overlap among BioProjects.

N. of DEGs (out of 6242)	N. of BioProjects (out of 18)	Gene Name
8	6	Islr2, Btg2, Col11a2, **Dusp1, Fos**, Slc13a4, **Tsc22d3**, Tshz2
29	5	Akap12, **Apod**, **Arc**, Bcas1, Camk2d, Cdr1, **Ddit4**, Dio2, Dock5, Egr4, **Etnppl**, Fosb, Gm20605, **Grin2b**, **Igf2**, Itih3, **Junb**, Mef2c, **Nfkbia**, **Npas4**, Otof, Pafah1b3, **Pcdha1**, **Pcdha12, Ptgds**, Rpl21, **Scn4b**, Tacr3, **Unc13c**
74	4	Acer2, Actn, Ahsa2, Ankfn1, Arhgap36, Baiap3, Bmp1,C4b, **Cck**, Chtop, Cmip, Col9a3, Cryab, Cyr61, Deptor, Dgkk, Dpm1-andp, **Drd1, Dusp6**, **Egr1**, Enpp2, Fam163b, Fam46a, **Fkbp5**, **Fmod**, Gdf1, **Gpr88, Htr2a, Ier2**, **Igfbp2**, Irs4, Kcnj2, Ldhd, **Magel2**, Mal, Man2c1, Mgst3, Mobp, **Necab1**, Nptxr, **Nr4a1, Nr4a3**, Pcdhga5, Peg10,Pik3r1m, Pisd-ps1, Prdx6, Rgs11, **Rn45s**, Rnf8-cmtr1, Robo3, **Rpl30**, Rps27, Rps27a, Rtn4r, Satb2, Scamp3, **Slc17a6**, Slc26a2, Spp1, Stab1, Stox2, **Tac1**, Thbd, Tmem158, Tmem252, Tmem254a, Trib1, Ucp2, Vwa5b1, **Vwf**, Zim1
364	3	see Supplementary Table 1
1451	2	see Supplementary Table 2

Each row shows the DEGs overlapping only in the number of BioProjects indicated in the second column. Genes highlighted in bold have been previously associated with stress in either humans, animal models or both.

**Table 4:** DEGs overlap among Configurations.

N. of DEGs (out of 6242)	N. of Configurations (out of 101)	Gene Name
1	14	Fos
1	13	FosB
2	12	Tsc22d3, Egr1
6	11	Npas4, Sgk1, Nfkbia, Btg2, Dio2, Dusp1
6	10	JunB, Cyr61, Arc, Arl4d, Nr4a1,Alas2
7	9	Tshz2, Vwf, Egr2, Egr4, Ddit4, Etnppl, Sik1
6	8	Prdx6, Pcdha1, Bcas1, Vwa5b1, Rps27, Apold1
11	7	Igf2, Hbb-b2, Camk2d, Txnip, Cck, Col11a2, Isrl2, Scn4b, Nr4a3, Baiap3, Trib1

Each row shows the DEGs overlapping only in the number of Configurations indicated in the second column.

**Fig. 4:**
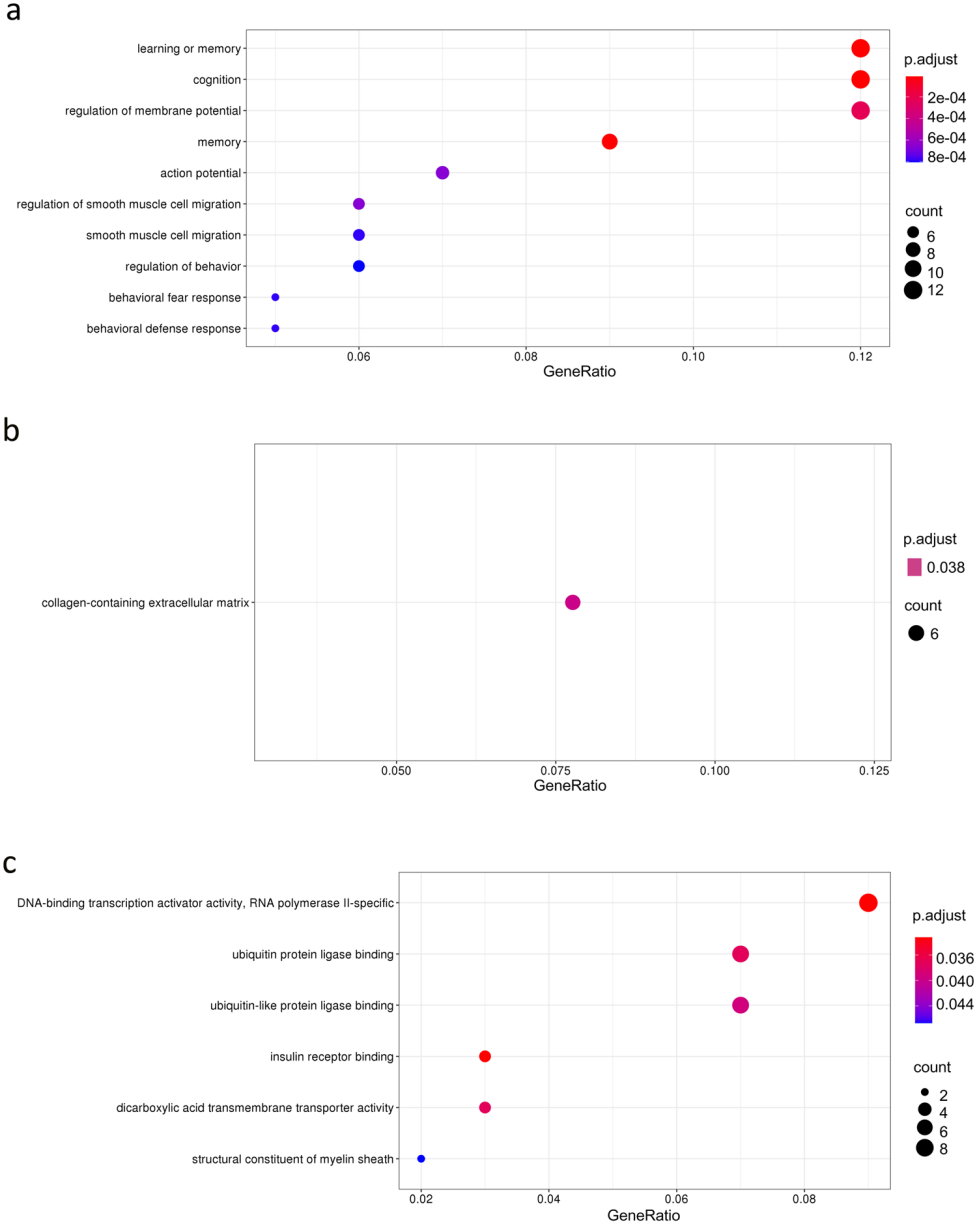
Analisis of enriched gene ontology (GO) terms for shared DEGs. The plots represent the first ten most significantly over-represented gene ontology terms in the biological process (**a**), cellular component (**b**) and molecular function (**c**) categories. For each analysis, significant DEGs overlapping across at least 4 BioProjects were used as input. The size of each dot represents the number of genes enriched in that term, while the color represents the corrected p-value. p.adjust < 0.05 was considered significant.

To gain insight into the biological processes and functions which were most represented by the DEGs in common to different stress conditions, we applied a functional annotation analysis with clusterProfiler, using the over-representation test with a hypergeometric distribution model^39^. Selecting as input the list of 111 genes that were significantly regulated by stress in 4 or more independent studies (Table 3), the gene ontology (GO) analysis revealed a strong enrichment for brain-specific and stress-related terms, as shown in Fig. 4. In the “Biological Process” group the highly significant terms were mostly related to regulation of behavior, such as learning and memory (p.adjust = 1.07 × 10^−5^) cognition (p.adjust = 1.21 × 10^−5^), regulation of membrane potential (p.adjust = 1.21 × 10^−5^), behavioral fear response (p.adjust = 0.00079) and behavioral defense response (p.adjust = 0.0008). Among the “Cellular Components” our selected DEGs were especially enriched for collagen-containing extracellular matrix (p.adjust = 0.038). Finally, the most represented terms in the “Molecular Function” category were DNA-binding transcription factor activity (p.adjust = 0.033), ubiquitin protein ligase binding (p.adjust = 0.033), insulin receptor binding (p.adjust = 0.037) and structural constituent of myelin (p.adjust = 0.047).

We associated the GO analysis to a protein-protein interaction (PPI) network analysis and a manually curated search of the literature in order to obtain a more defined picture of the processes affected by these genes. As reported in Table 3, some of these genes had previously been associated with stress or glucocorticoids receptor (GR) regulation while others, such as *Islr2, Btg2, Col11a2, Slc13a4, or Tshz2* can be considered novel candidate stress-associated genes. We found a large enrichment in DNA binding transcription factors or RNA binding proteins: *Dusp1, Fos, Igf2, Arc, Btg2, Chtop, Egr4, JunB, Mef2c, Npas4, Sat2b, Egr1, Nr4a1*. The genes *Ddit4, Nfkbia, Cmip* are related to the Nf-kB pathway while *Coll11a2, Ptgds, Fmod, Pcdhga5, Spp1, Vwf* code for extracellular matrix proteins.

**Fig. 5:**
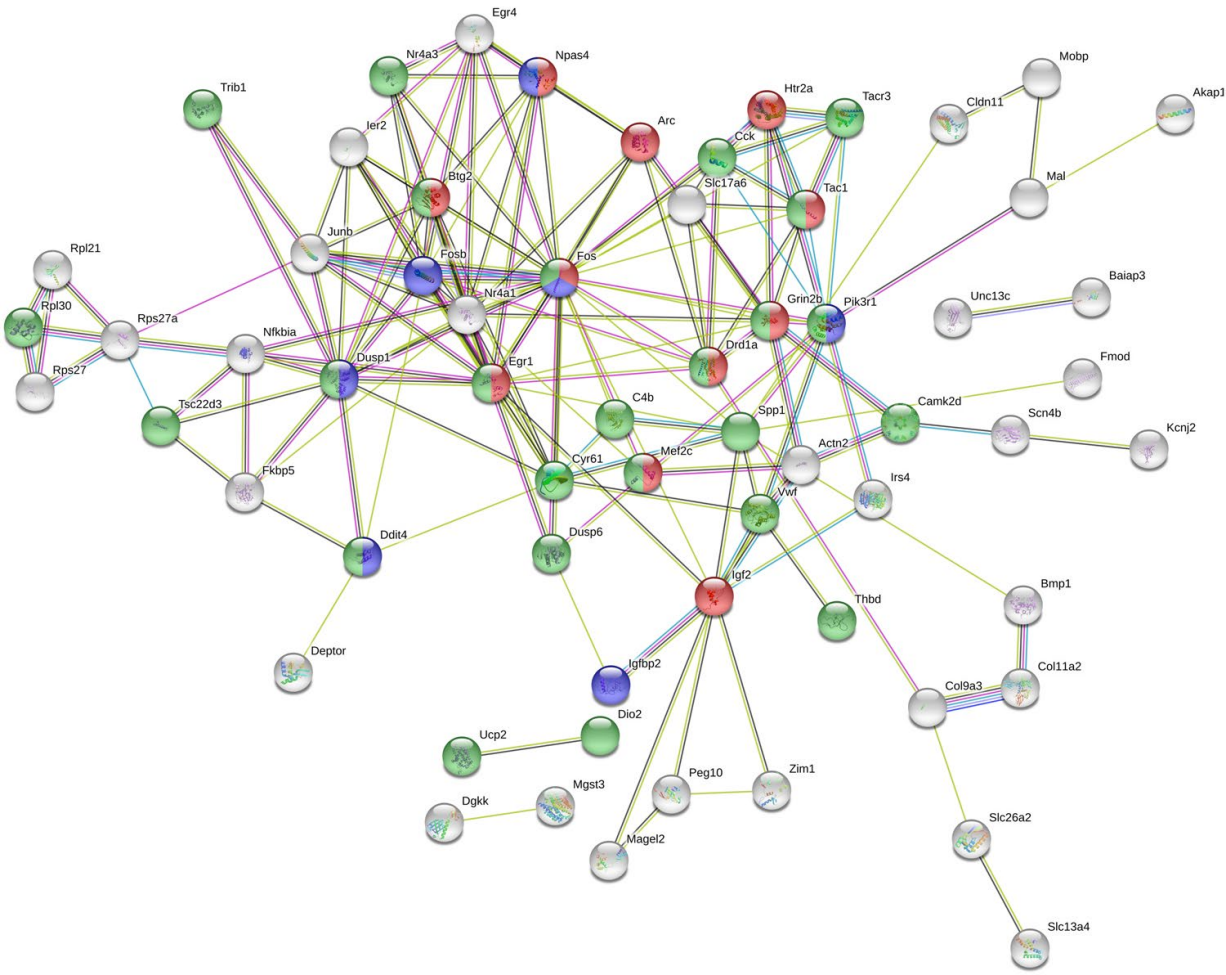
Protein-protein association network for shared DEGs. The plot represents the protein-protein interaction network for the 111 genes significantly different in at least 4 BioProjects. The network nodes represent proteins. Colored nodes highlight proteins in the GO biological process categories response to glucocorticoids (blue), response to stress (green) and learning and memory (red). The network edges represent protein-protein associations, known from curated databases (light blue), experimentally determined (pink), or predicted from gene co-expression (black), co-occurence (blu), or from textmining (yellow). Only connected nodes are shown in the network.

The PPI network analysis with STRING^40^ (Fig. 5) highlighted a statistically significant association among the top 111 DEGs (PPI enrichment p-value: <1 × 10^−16^). This means that the proteins have more interactions among themselves (167 edges, from known and predicted interactions) than what would be expected for a random set of proteins of similar size, drawn from the genome (expected number of edges: 45), indicating that the proteins are at least partially functionally connected. The PPI network was significantly enriched for several GO terms, including response to stimulus (63 genes out of 6616 belonging to this category, FDR = 6.35 × 10^−8^), response to glucocorticoids (blue nodes in Figs. 5, 8 genes out of 159, FDR = 7.22 × 10^−5^), response to stress (green nodes in Fig. 5, 30 genes out of 2899, FDR = 0.00086) and learning or memory (red nodes in Fig. 5, 12 genes out of 286, FDR = 2.79 × 10^−6^).

To evaluate the impact of factors that were very heterogeneous in our dataset, such as the type or duration of stress, or the brain region analyzed, we performed additional analysis on the DEGs profile of subsets of the whole dataset.

**Fig. 6:**
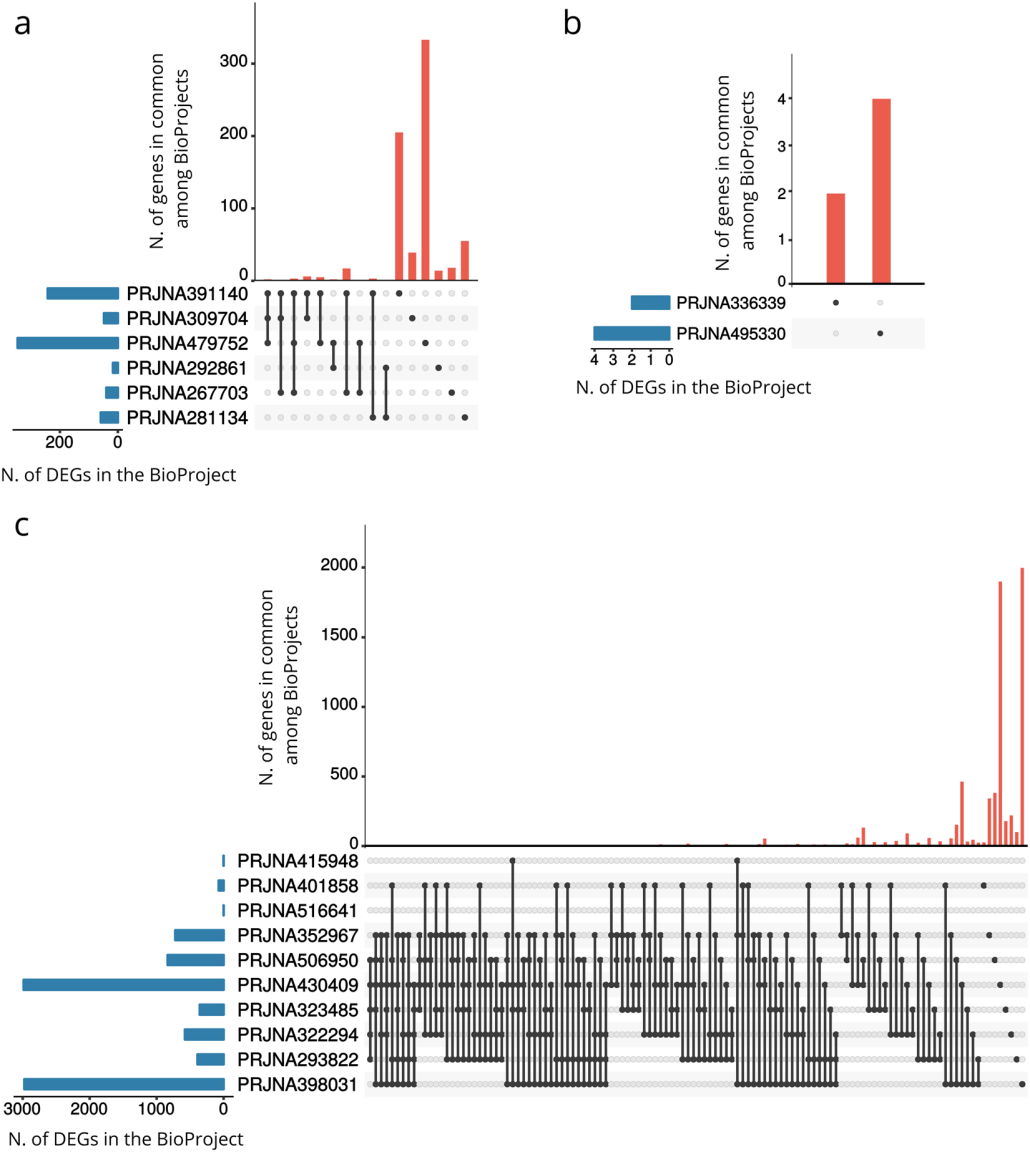
Comparison of acute, subchronic and chronic stress protocols. The upset plots show the intersection of differentially expressed genes across subsets of BioProjects that used acute (**a**), subchronic (**b**) and chronic (**c**) stressors. The horizontal bar graph on the left shows the total number of DEGs for each BioProject. The red upper bar graph shows the number of DEGs for each overlapping combination. Black connected circles indicate which BioProjects are involved in each intersection.

**Fig. 7:**
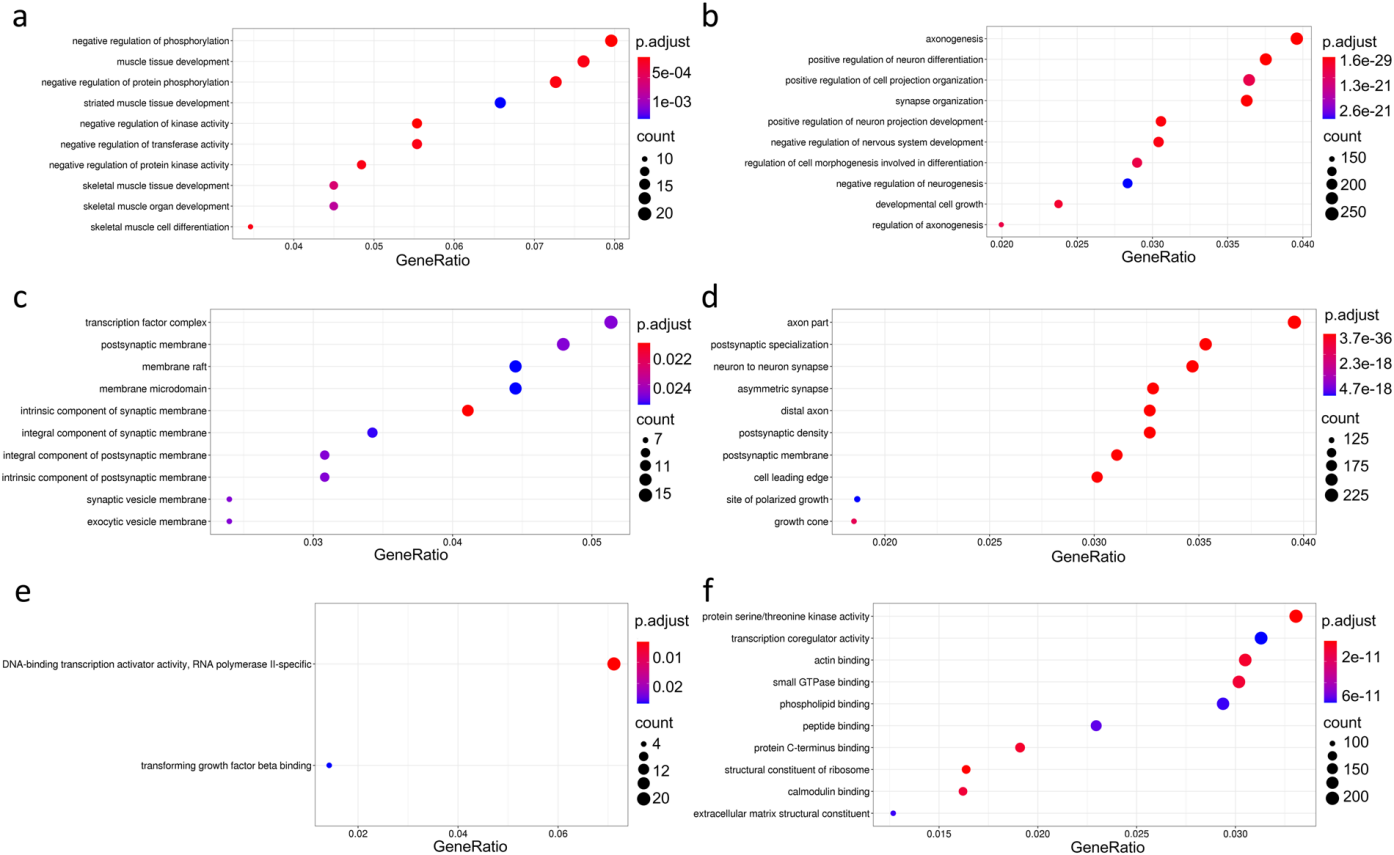
Analysis of enriched gene ontology (GO) terms for DEGs at short and long time points after stress. The plots represent the first ten most significantly over-represented gene ontology terms in the biological process (**a**,**b**), cellular component (**c**,**d**) and molecular function (**e**,**f**) categories, for the list of genes differentially expressed after a short (<=24 hours) (a,c,e) or long (>24 hours) (**b**,**d**,**f**) delay after the end of stress. The size of each dot represents the number of genes enriched in that term, while the color represents the corrected p-value. p.adjust < 0.05 was considered significant.

First we explored the data based on the duration of the stressor. As shown in Fig. 6a–c, chronic stress induced a much higher number of DEGs compared to acute and subchronic stress. The extent of DEGs overlap among chronic stressors was also greater compared to acute stressors. No overlap was observed between the two studies involving subchronic stress, which were also showing a very small number of DEGs. Then we compared studies that investigated short-term effects of stress (<=24 hours) as opposed to more long-lasting (>24 hours), performing a GO analysis on the genes differentially expressed in the two conditions. It should be noted that in our dataset, the majority of the BioProjects using a short delay between end of stress and brain dissection were also using an acute stressor, as shown in Table 2. As shown in Fig. 7a,c,e, when we analyzed the DEG profile at the earlier time points, we found a strong enrichment for terms related to negative regulation of phosphorylation and kinase activity, as well as muscle development in the “Biological Process” category, for terms related to the synaptic membrane in the “Cellular Component” group, and for transcription activation in the “Molecular Function” category. The GO analysis of the genes differentially regulated at longer intervals after stress (Fig. 7b,d,f) highlighted terms related to regulation of neurons development, such as axonogenesis, projection development, synapse organization, neurogenesis and differentiation in the “Biological Process” category, again terms mostly related to the synapse in the “Cellular Component”, and finally several protein binding terms in the “Molecular Function” group.

Next, we refined our analysis, searching for DEGs shared among configurations that used comparable stress protocols, in terms of type of stress and interval between the RNA-seq analysis and the end of stress. Four configurations in our dataset (one in PRJNA309704 and three in PRJNA391140, see Table 2) investigated the effect of acute (30 min) restraint stress in the HIPP, using a comparable delay (within 1 hour) and the same mouse strain. We found 188 DEGs, of which 32 shared between 2 or more configurations. *Fos* was significantly upregulated in all four configurations. *Slc2a1, Dio2, Errfi1, Ddit4, and Tsc22d3* were significantly upregulated, while *Kcnj2* was downregulated, in three configurations spanning both BioProjects.

Electric foot shock was used in four independent studies, but we found a high variability in the pattern of gene expression between them. We found no overlap when comparing quite similar configurations such as those in PRJNA267703 and PRJNA292861 (Fig. 6a and Table 2), although both of them shared a few DEGs (*Btg2, Fosb, Malat1, Prdx6, Sox18, Tm6sf2*) with PRJNA479752, that investigated the AMY instead of the HIPP.

Our dataset included a prominent number of BioProjects that investigated the transcriptomic profile after chronic social defeat. When comparing these six BioProjects, we found a greater overlap, with 113 DEGs in common among at least three BioProjects (Supplementary Table 3), of which *Slc13a4* differentially regulated in five studies out of six; and *Arhgap36, Baiap3, Cck, Cldn11, Fam163b, Grin2b, Igf2, Irs4, Megf6, Otof, Ptgds, Rn45s, Robo3, and Scn4b* in four studies out of six. We used this list of 113 genes as input for a GO analysis. The most over-represented terms in the “Biological Processes” group were related to regulation of membrane potential (p.adjust = 0.00017) and neurotransmitter secretion (p.adjust = 0.00017), followed by signal release from a synapse (p.adjust = 0.0018), learning or memory (p.adjust = 0.0018), neuron/axon ensheathment (p.adjust = 0.0018), multicellular organism response to stress (p.adjust = 0.0018) and fear response (p.adjust = 0.0018). In the “Cellular Components” category we found extracellular matrix (p.adjust = 0.0002) and many axon-related terms, such as axon part (p.adjust = 0.001), distal axon (p.adjust = 0.0026), myelin sheath (p.adjust = 0.0026) and presynaptic membrane (p.adjust = 0.0027), while extracellular matrix structural constituent (p.adjust = 2.48 × 10^−5^) and structural constituent of myelin sheath (p.adjust = 0.0006) were the significant terms in the “Molecular Function” group.

**Fig. 8:**
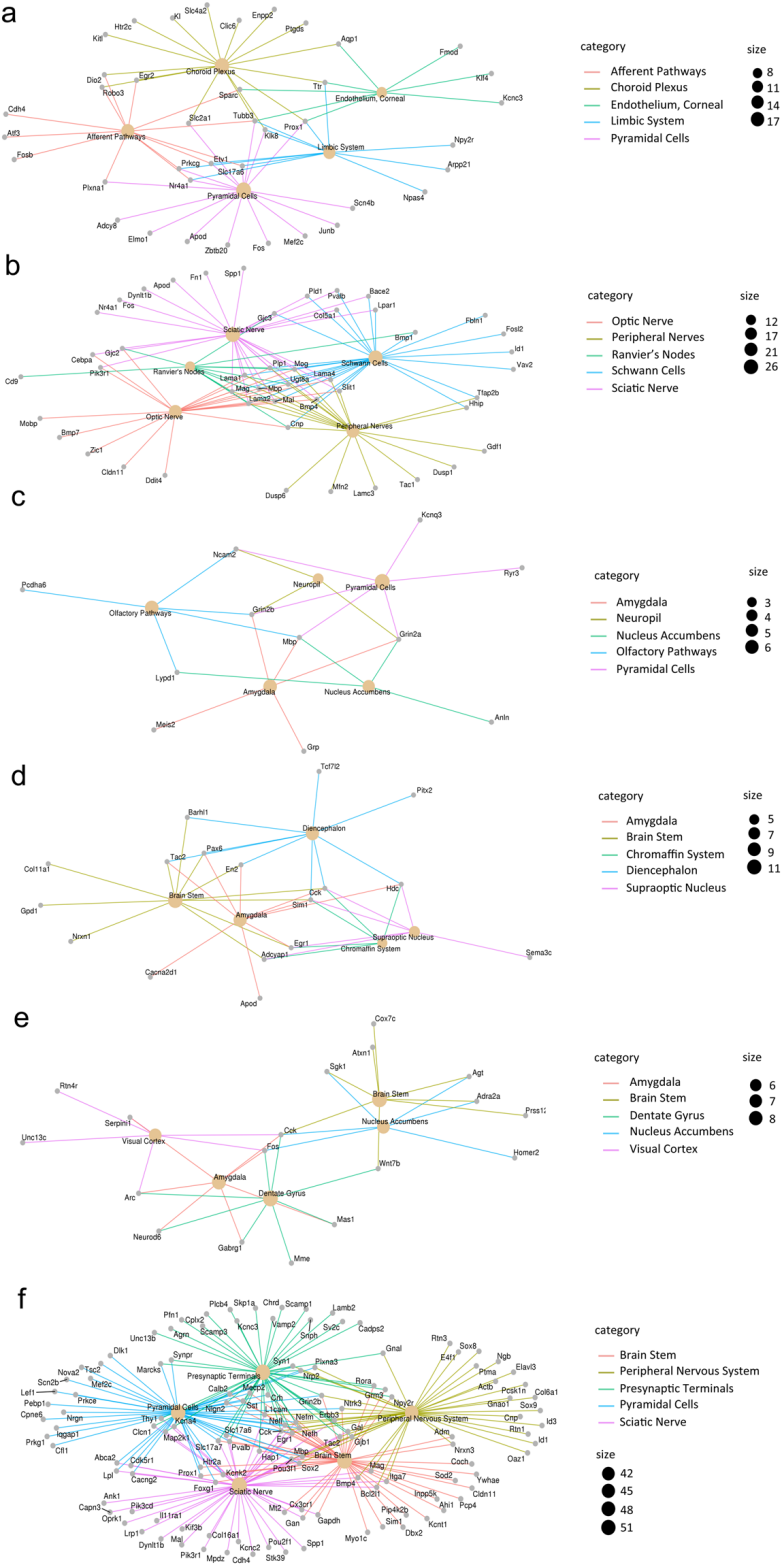
The plots represent the first five most significantly overrepresented anatomy terms according to MeSH and the associated annotated genes for (**a**) hippocampus, (**b**) prefrontal cortex, (**c**) amygdala, (**d**) ventral tegmental area, (**e**) nucleus accumbens, (**f**) bed nucleus of the stria terminalis. The size of each node (anatomy term) represents the number of DEGs enriched in that anatomy term.

To investigate regional signatures of stress, we studied gene expression patterns separately for each brain area, by comparing all the BioProjects (or selected configurations within a BioProject) that investigated a particular region. For each region we obtained a different set of DEGs, and observed a limited intersection among the different studies (Table 5 and Supplementary Tables 4, 5, 6, 7). The maximum number of BioProjects sharing at least one DEG was four out of ten for the HIPP; 3 out of 7 for the PFC; 3 out of 6 for the NACC; and 2 out of 4 for the AMY (Table 5). Then we asked if, for each region, DEGs were enriched for cell type-specific genes. To this aim, we assessed the enrichment of the stress-related transcriptomic profile of each region for anatomy-associated terms, based on MeSH annotations using ClusterProfiler^39^ (Fig. 8). For the HIPP, the second most significant term was pyramidal cells (p.adjust = 1.44 × 10^−8^), followed by limbic system (p.adjust = 2.20 × 10^−8^), but the first significant term was choroid plexus (p.adjust = 6.32 × 10^−10^) (Fig. 8a). PFC DEGs analysis revealed enrichment for Schwann cells (p.adjust = 1.72 × 10^−15^), sciatic nerve (p.adjust = 2.43 × 10^−15^) and Ranvier’s node (p.adjust = 1.82 × 10^−11^) (Fig. 8b). When we analyzed the AMY data, the term amygdala came out as the second most significant (p.adjust = 0.0005). This term presented extensive overlap with other terms that were significantly enriched, such as pyramidal cells (p.adjust = 0.00053) and nucleus accumbens (p.adjust = 0.0018) (Fig. 8c). VTA analysis returned the brain stem as the most enriched term (p.adjust = 3.18 × 10^−8^) (Fig. 8d). NACC analysis resulted in the significant term nucleus accumbens (p.adjust = 0.00034), but closely preceded by visual cortex (p.adjust = 0.00012), amygdala (p.adjust = 0.00021) and dentate gyrus (p.adjust = 0.00021) (Fig. 8e). Finally, for the BNST the MeSH-based enrichment analysis returned many significant terms, but mostly non specific for BNST (Fig. 8f).

**Table 5:** DEGs shared among BioProjects divided by brain region.

Brain region	N. of DEGs/Total	N. of BioProjects/Total	Gene Name
Hippocampus	2/3204 21/3204 159/3204	4/10 3/ 10 2/10	Camk2d, Tshz2 Cobl, Hspa1a, Mef2c, Ptgds, Satb2, Slc17a6, Unc13c, Akap12, Bcas1, Cdr2, Col9a3, Dusp1, Fam46a, Fos, Islr2, Junb, Kcnj2,Scn4b, Slc17a6, Tmem252, Tnfrsf25, Tsc22d3 see Supplementary Table 4
Prefrontal cortex	24/3553 204/3553	3/7 2/7	Cldn11, Dock5, Dpm1-adnp, Fbln1, Fkbp5, Hhip, Islr2, Mrc2, Pcdha12, Pcdha1, Pcdhga3, Pde8a, Pisd-ps1, Plp1, Rpl21, Rpl30, Slc26a2, Vwf, Btg2,Col8a2, Ddit4, Dusp1, Etnppl, Islr2, Slc47a1, see Supplementary Table 5
Nucleus Accumbens	3/877 76/877	3/6 2/6	Alas2, Arc, Npas4 1700020I14Rik, Adra2a, Basp1, C130074G19Rik, C1ql3, Cnih3, Cox7c, Crtac1, Efhd2, Fam163b, Fndc9, Galnt9, Golga7b, Homer2, Igfn1, Ipcef1, Kcnc4, Mast1, Mgst3, Mir6236, Necab3, Neurod6, Npas4, Nptx1, Nptxr, Olfm1, Ppm1e, Prss12, Prss23, Ptpn3, Rhou, Rtn4r, Seripini1, Sgk1, Sidt1,Stx1a, Syt13, Wnt7b, Agt, Ankrd33b, Arl10, Asb4, Atxn1, Brinp1, Dgkk, Dpm1-adnp, E130012A19Rik, Etnppl, Fam198b, Fos, Gabrg1, Hba-a2, Igsf1, Itih3, Kcnv1, Lhfp, Lrig1, Lrrc1, Magel2, Mas1, Mgst3, Mme, Msmo1, Necab1, Ntm, Osbp2, Pafah1b3,Pcp4, Plch2, Plcxd2, Rasgef1c, Rasl10a, Scml4, Slc6a11, Stac, Strn, Sv2b, Syt2, Tmem130, Tmem25, Tmem255a, Unc13c, Vwa5b1, Wdr6
Amygdala	35/644	2/4	Actn2, Anln, C1ql2, Cdkl5, Cntnap5b, Cryab, Dgkh, Filip1, Fos, Gpr26, Gpr6 Grin2a, Grin2b, Grp, Itgb4, Kcnq3,Kctd16, Lypd1, Malat1, Mbp, Meis2, Miat, Ncam2, Nkain3, Pcdha6, Plin4, Prdx6, Ryr3, Slc13a4,Tacr3, Tcea1, Tceal5, Tceal6, Trhr, Trpm3
Bed Nucleus of the Stria Terminalis	8/1009 1001/1009	2/2 1/2	Baiap3, Cnp, Cpne6, Fa2h, Flt3, Nefl,Otof, Rec8 see Supplementary Table 6
Ventral Tegmental Area	59	1/1	see Supplementary Table 7

For every region, each row shows the DEGs (out of the total number of DEGs for that brain region) overlapping only in the number of BioProjects indicated in the third column (out of the total number of BioProjects investigating that region).

## Web Portal Description

The results of the transcriptomic re-analysis were systematized in an open-source web portal available at the following link: http://hpc-bioinformatics.cineca.it/stress_mice/.

Stress mice Portal is a free and curated web server able to query through different criteria all of the results obtained from this data analysis. The web portal is made of 7 sections and 13 subsections: the “Home Page”, the “Search” utility, the “Dataset” section and the “Statistics”, “Download”, “Contact” and “Help” pages.

Here is a more detailed description of each section.

- **Home**. In the homepage the user is provided with a quick overview of the web portal.
- **Search**. This utility allows several searching options to browse and mine the results obtained from the re-analysis of the various BioProjects (Table 6). The results of the queries are presented in the form of a paginated table containing DEGs which satisfy the query parameters and data can also be downloaded in tabular format.

**Table 6:** Example of possible queries on Stress Mice web portal.

Search by	Example Question	Example of query with default values
Genes shared among BioProjects	Which are the DEGs shared among BioProjects containing samples from AMY?	Select BioProjects ‘ALL’, Criterion1 ‘Subregion = = “amy”‘
DEGs	Which are the the DEGs under a particular stress treatment for e specific BioProject?	Select the desired FC, the desired p-value and q-value (at least 0.05), BioProject ‘PRJNAXXX’, Stress protocol (e.g. “CFC”)
Gene	Is GeneX differentially expressed? In which BioProjects and configurations?	Insert Gene name and leave default values.
Brain region	Which genes are differentially expressed in BNST?	Select brain region = = ‘BNST’

The default values are log2(FC) > = 0.38, p-value < = 0.05, q-value < = 0.05.

### Search by BioProject or search for genes shared among BioProjects

By selecting only one BioProject ID and the variable to test (e.g., type of stress), the computation of DEGs is executed within the study. On the contrary, by selecting “ALL” the BioProjects and choosing one or more variables to test, it will be possible to obtain the overlap of the results between multiple studies.

### Search for DEGs

This utility allows for an “on-the-fly” computation of DEGs in a single BioProject of interest. A gene is declared differentially expressed if an observed difference or change in read counts or expression levels between two experimental conditions is statistically significant.

We suggest leaving p-value and q-value fixed to 0.05 and selecting as minimum linear fold-change any value ranging from 1.3 to 4 with step = 0.1. Graphs for these settings, in fact, have been pre-computed and are immediately available on the web page after the search button is clicked; in case users need custom values, results might take 1–2 minutes to show up.

Obviously, when computing differentially expressed genes, the number of pairwise contrasts can vary depending on the metadata of the BioProject of interest (e.g. there can be multiple time points or multiple stress protocols for the same BioProject). Therefore, we set up a dynamical drop down menu where the criteria of choice are compatible with the specific BioProject of interest.

Moreover, since there can be more than one configuration analysis for a single BioProject (e.g. you can compute DEGs comparing samples with different stress protocols and leave blank the remaining criteria of choice of samples) the final results will regard all the possible combinations of configurations.

### Search by gene

The user can query the database by one or more gene symbols of interest. The resulting table shows the configurations and the corresponding BioProjects in which the genes of interest are differentially expressed in the stressed samples compared to the control ones. The table contains links to external resources, e.g. by clicking on the gene symbol the user will be redirected to the NCBI web page specific for that gene. Moreover, by clicking on the configuration ID, it is possible to compute and download graphics such as heatmaps, volcano plots and Gene Set enrichment analysis results for the corresponding configuration.

### Search by brain region

This search page is dedicated to those users who wish to explore a particular area of the brain, by selecting one of the following brain regions: “AMY, PG, BNST, VTA,PFC, HIPP, HT or NACC. For example, by selecting “AMY”, the resulting table will show DEGs among all the BioProjects containing samples derived from AMY region and returns a summary explaining how many genes result differentially expressed and in which BioProjects and configurations.

- **Dataset**. This page gives an overall description of the input dataset used in this study. Here we report detailed information for each BioProject involved in the research, including the number of samples, the sequencing platform, a link to the original paper, and so on. The “Dataset Phenotypic data” page is a deeper graphical overview of the different experimental protocols originally applied to the samples of each BioProject.
- **Statistics**. This section allows a visual inspection of the results. Four sub-menus are available: DEG statistics by BioProject, by Configuration or by Brain Region. In the fourth sub-menu, named “DEGs intersections across different BioProjects”, it is possible to dynamically build a Venn diagram showing the results of the analysis in terms of the number of DEGs found in each BioProject or the number of DEGs shared among the selected BioProjects.
- **Download**. From this panel it is possible to download all the processed data described within this article.

## Discussion

We selected and re-analyzed the raw RNA-seq data from 18 independent BioProjects downloaded from SRA. The dataset presented many inter-study differences, both in terms of technical (e.g., sample preparation, library protocols, sequencing platform) and biological conditions. All studies were conducted on mice, but they differed for several aspects, including: strain, age and sex of subjects, type of stress, interval between stress and transcriptomic analysis, brain region analyzed. Given this heterogeneity, although all data were re-analyzed with the same transcriptomic pipeline, in order to perform differential expression analysis we curated 101 unique experimental comparisons (stress versus control), only between samples belonging to the same BioProject.

An increasing literature shows that different stress conditions induce distinct gene expression profiles in the brain^11–16^. The behavioral and physiological response to stress is fine tuned by a complex cascade of activation and feedback-regulation of many systems, including the autonomic and the central nervous systems, the immune and the metabolic systems^3–6^, thus it is conceivable that factors such as the duration and intensity of stress, could affect partially different sets of genes^13,41^. The sex of the subject is well know to influence the response to stress^15,24,32^. Moreover, due to the highly dynamic nature of the stress response, the time from stress^33^ or the brain region analyzed^7,16,26^ are other important aspects to take into consideration. Finally, genetically inbred strains of mice are characterized by a different susceptibility and neurobiological reaction to stress^42–44^.

Coherently with the above literature, although we observed many significant overlaps of DEGs between pairs of BioProjects, overall the number of DEGs shared among more than two different Bioprojects was relatively low. Nonetheless, comparison of transcriptome changes across different studies could help to identify common underlying biological processes. A GO analysis of the 111 genes significantly regulated in at least 4 BioProjects pointed to a strong regulation of genes involved in cognitive functions, particularly learning, memory, and fear response. These results are particularly interesting in light of a wide set of evidence showing that altered plasticity and cognitive abilities are a hallmark of stress-related disorders^2,3,9,45^. Interestingly, the PPI network analysis pointed out a relevant functional connection among these genes, showing a significant association among their protein products, and a significant enrichment for terms related to stress and cognition.

Indeed, by analyzing in depth the known function of the top 111 common DEGs we found many DEGs with similar biological roles. Many of these genes were associated with stress or GR regulation from previous studies, either in animal models or humans or both, as in the case of *Fkbp5*. The FKBP5 protein is a co-chaperone of GR, that prevents translocation of GR to the nucleus thus modulating its function^46^. Gene association studies have shown that polymorphisms in *Fkbp5* predict adult PTSD or depression onset associated with childhood traumatic stress^46,47^.

A high number of the top-regulated genes are DNA binding transcription factors or RNA binding proteins (*Dusp1, Fos, Igf2, Arc, Btg2, Chtop, Egr4, JunB, Mef2c, Npas4, Sat2b, Egr1, Nr4a1*). Many of these genes, such as *Fos, Egr1, Egr4, JunB, Arc, Dusp1* are rapidly induced in the brain by a variety of stimuli, while repeated stimulation has a repressive effect, thus their expression is used as an index of neuronal activation^48^. In addition, these genes have also been involved in stress reactivity, as they are induced by acute stress and often reduce their expression upon repeated exposure to a stressor^49,50^. Mutual inhibition through direct interaction has been shown *in vitro* between GR and either c-Fos or c-Jun, two immediate early genes (IEGs) that dimerize to form the AP-1 complex, which is an important transcriptional regulator^51,52^. *Arc* is a synaptic plasticity-associated IEG whose expression is also induced by stress^50^. Indeed, our results confirm differential regulation of these genes in a high number of BioProjects, with *Fos* being the DEG varied in the highest number of configurations, closely followed by other IEGs such as *FosB, Egr1, Egr2, Arc, JunB and Egr4*. Interestingly, in our datasets Fos followed a very predictable trend, being consistently over-expressed after acute stressors (restraint, forced swim and electric footshock), but down-regulated after chronic stress (social defeat and variable stress).

Among the selected genes, we observed three factors related to the Nf-kB pathway (*Ddit4, Nfkbia, Cmip*) that is important in the control of inflammation and has been linked to stress-induced development of psychiatric disorders^53,54^. *Coll11a2, Ptgds, Fmod, Vwf, Pcdhga5 and Spp1* are genes of the extracellular matrix, that in the brain is important not only for maintaining, but also for remodelling neural architecture, thus playing a role in stress-induced structural plasticity^55–57^.

Alongside already described stress-regulated genes, we identified a number of interesting novel candidate genes that had not been previously linked to stress. For example, *Islr2* codes for a protein required for axon extension during neural development, when it has an essential role in the establishment of forebrain connectivity^58^, but with unknown functions in adults. In adult mice *Islr2* is selectively expressed only in neurons and testis^59^. *Bcas1* is a gene abundantly expressed in the brain. Its function is still unclear, but it has been associated with myelination^60^. Interestingly, among the top candidates differentially expressed in 5 different BioProjects we identified another gene involved in myelination, *Akap12*^61^. Although these two genes have not been linked to stress previously, myelin reduction and regulation of myelin genes has been consistently observed after chronic stress^30,62,63^.

Finally, two other potentially relevant novel genes are *Mef2c* and *Sat2b*. Both genes are transcriptional regulators involved in long term memory and synaptic plasticity^64,65^, and have recently been identified as genetic risk loci for schizophrenia^65,66^.

Considering the duration of stress, we observed that acute and chronic stressors have quite different effects on gene expression in the brain, both in terms of overall number of DEGs and DEGs identity. The GO analysis of genes modulated at short time points after the end of stress, revealed a significant enrichment for genes related to regulation of several enzymatic activities, transcription factors and synaptic membrane. At longer time points after stress, the functional enrichment analysis pointed out many terms pertaining to more structural changes, such as axogenesis, synapse organization and projection development. This is coherent with studies showing that the initial response to stress involves rapid changes in neuronal function, and it is followed by structural plasticity events, leading to alteration of dendritic spine dynamics and rearrangement of local synaptic circuits, especially after chronic stress^3,6,67^.

When we investigated the gene expression pattern separately for each brain region, we found again limited overlap among different BioProjects, as well as among brain regions. The analysis of the anatomic specificity of the DEGs for each brain area, conducted looking at the overall enrichment for anatomy-associated terms, suggests that for HIPP and AMY stress affected many region-specific genes. Indeed HIPP DEGs were particularly enriched for the term pyramidal cells, the principal cell type of the hippocampus, and limbic system. It is important to note that our analysis indicated that HIPP samples were also enriched for DEGs expressed in the adjacent choroid plexus tissue, that could be due to a contamination of hippocampal tissue with choroid plexus during brain dissection^68^. AMY DEGs were significantly enriched for the MeSH term amygdala, but also for terms such as nucleus accumbens, pyramidal cells, and olfactory pathways, that have direct anatomical connections with the amygdala. Although we did not find specific PFC terms, PFC DEGs analysis revealed a strong enrichment for nerve-related terms, that could reflect the high short and long range connectivity of this cortical region^69^. The most enriched term from VTA analysis, brain stem, is coherent with the localization of this region. Finally, analysis of NACC and BNST DEGs did not highlight a relevant anatomical-specificity of the genes modulated by stress in these regions.

In conclusion, these results suggest that a comparative re-analysis of brain-wide large-scale gene expression data could help identify stress-related functional pathways and novel candidate stress-responsive genes, but taking into account the different attributes of different stress protocols and subjects.

In the future, continued integration of novel datasets within this database will allow us to extend and test the reliability of these findings. The web portal that we created provides a dynamic and freely available web platform for obtaining information on genes related to different kinds of stressors and their association with other relevant parameters, such as brain region and sub-region, strain, sex, or stress susceptibility. Stress Mice Portal is very versatile and interactive and will allow researchers interested in stress to explore the data, as well as to easily re-use the results for further analysis and comparisons.

## Methods

### Data retrieval

Selecting interesting BioProjects consists in inspecting each and every BioProject according to some criteria (its size, the number of samples contained, and so on). In order to build the whole dataset, we queried SRA portal using the following key terms: “mouse stress brain”, “mouse stress hippocampus”, “mouse stress amygdala”, “mouse stress cortex”, until April 2020. We selected only BioProjects containing RNA-seq SRA-experiments that met the following criteria: I) have an associated publication in order to retrieve relevant metadata; II) include at least six samples with a minimum of 3 biological replicates per condition; III) include a non-stressed control group; IV) include samples from wild-type and drug-free animals; V) include samples from at least one brain region or sub-region. These queries resulted in 18 BioProjects responding to these criteria, for a total of 751 samples.

Not to mention, additional metadata regarding each Biosample in the SRA-experiment (e.g., information about the sex of the sample, the sample tissue, the age of the sample, etc.) are not directly available in the same web page of the experiment and a new page needs to be opened for each sample for retrieving such information. Performing such selection manually might be a very complex and error-prone task.

To overcome these obstacles, we developed a custom python script in order to automatically download metadata from NCBI/SRA database and dynamically collect them (Tables 1 and 2 summarize all the retrieved information).

Sometimes, in case of lack of information (missing values of attributes), it was necessary to retrieve metadata in the supplementary material of the associated PMID or by manually scanning other databases (e.g. GEO). The data-sheet of metadata for each BioProject is available on Mice Stress Portal and downloadable from the page “Dataset overview” http://hpc-bioinformatics.cineca.it/stress_mice/dataset_overview under the column “Phenodata download”.

### Bioinformatics analysis

The whole dataset was composed of both Single-End and Paired-End RNA-seq samples, for a total of about 2,5 TB of input raw data. The transcriptomic pipeline, shown in Fig. 1 and described more in detail in the Supplementary Information, was applied to each single BioProject. Samples from different BioProjects were never combined together in the transcriptomic analysis.

Raw reads were obtained in FASTQ format and were quality-assessed using FastQC program http://www.bioinformatics.babraham.ac.uk/projects/fastqc. Terminal low quality bases and adaptor sequences were trimmed off using Trimmomatic utility^70^. Clean reads were aligned against UCSC reference genome *mm10* obtained from Illumina Genome website using the splice-aware read mapper HiSat2^71^. BAM files obtained from read alignment were further processed with Stringtie in order to assemble known transcripts^72^. HTSeq (https://htseq.readthedocs.io, version 0.6.1) was then used to quantify raw read counts.

### Differential expression analysis

Our aim was to investigate the effect of different types of stress on the brain transcriptomic response, taking into account also potential influences from other conditions such as the brain region analyzed, the strain, the sex of the subjects, or the delay between stress and brain dissection. For this reason, calculation of differential expression of genes was implemented by comparing samples that differed only in the exposure/non exposure to stress, but were matched with respect to all other variables. In order to define the correct comparison groups, first we carefully examined each BioProject to identify all the independent (or grouping) variables with two or more levels. Then, within each study we carried out comparisons between stressed and non-stressed animals separately for each level of the independent variables other than “stress exposure”. For example (see Table 2) in the dataset PRJNA398031^24^, besides the variable “stress exposure” (two levels: “chronic variable stress” vs. “control”) we identified two more grouping variables: “brain region” (two levels: “PFC” or “NACC”) and “sex” (two levels: “male” or “female”). Thus, we compared stressed and non stressed samples in this BioProject separately for PFC males, PFC females, NACC males, NACC females, carrying out a total of four comparisons.

Overall, in the entire dataset we identified 101 direct comparisons, that we termed “configurations” (see Table 2 for a complete overview of experimental variables and configurations).

Samples exposed to stress in each BioProject were always compared to the internal control samples belonging to the same BioProject.

A custom python script (available at: http://hpc-bioinformatics.cineca.it/stress_mice/scripts/) was used in order to prepare all the possible comparison configurations (n = 101) and R packages DESeq2^73^, clusterProfiler^39^ and AnnotationHub^74^ were subsequently employed to ascertain lists of differentially expressed genes (DEGs) and to obtain biological-term classification and the enrichment analysis of gene clusters. For the GO analysis with clusterProfiler, the size of annotated gene sets for testing was limited to 5-700 genes as lower/upper limit. STRING Database (v.11.0) was used to perform the PPI network analysis (https://string-db.org/)^40^ and Intervene Shiny App for visualization of DEGs intersections (https://intervene.shinyapps.io/intervene/)^75^. STRING assumed the whole genome as statistical background for the enrichment analysis. The significance of the overlap of DEGs between pairs of BioProjects was evaluated by one-tailed Fisher’s exact tests. P-values were corrected for multiple testing using the FDR. Overlaps showing FDR corrected p-values lower than 0.05 were considered significant. The background number of genes for the Fisher’s test was set to 39,736.

### Integrating the dataset with new BioProjects

The 18 selected BioProjects are in compliance with the filtering criteria described in the subparagraph “Data retrieval”. The authors will periodically check the literature and SRA for new BioProjects of interest, in order to expand the dataset, enrich the database and enrich the analysis. Any suggestion about new BioProjects of interest will be considered by the authors, who will carefully check the eligibility of the individual studies based on the criteria defined in the “Data retrieval” section. For suggesting the integration of new BioProjects in the database, users can refer to the contact page of the web portal (http://hpc-bioinformatics.cineca.it/stress_mice/contact).

## Supplementary information

Supplementary Table 1

Supplementary Table 2

Supplementary Table 3

Supplementary Table 4

Supplementary Table 5

Supplementary Table 6

Supplementary Table 7

Supplementary Information

## Data Availability

The datasets obtained and described within this article are freely downloadable at the following URL: http://hpc-bioinformatics.cineca.it/stress_mice/download. Normalized expression count tables, differential expression tables, differentially expressed genes for each configuration and relevant sample metadata were deposited on the repository Figshare (10.6084/m9.figshare.c.4860843)^76^.

## Code availability

The bioinformatics analysis pipeline, with a detailed list of command lines, was deposited on the repository Figshare (10.6084/m9.figshare.c.4860843)^76^.
