# Supplementary material for: A gene expression atlas for different kinds of stress in the mouse brain
Source: Sci Data. 2020 Dec 16;7:437. doi: 10.1038/s41597-020-00772-z (PMC7744580; doi:10.1038/s41597-020-00772-z)
Supplement: Supplementary file 8 — Supplementary Information [file 41597_2020_772_MOESM8_ESM.pdf]

Supplementary Information

List of commands of the applied transcriptomic pipeline:

As shown in figure 1 the pipeline works at "Sample level" from Step 1 to Step 8.

Step 1: Data Downloading

Single End) fastq-dump \$SAMPLE
Paired End) fastq-dump --split-files \$SAMPLE

Step 2 and 4: Quality check before and after read trimming

fastqc \$SAMPLE
fastqc \$SAMPLE.trim.fastq

Step 3: Read Trimming

Single End) java -jar trimmomatic-0.36.jar SE -threads 36 -phred33 \$SAMPLE.fastq \$SAMPLE.trim.fastq ILLUMINACLIP:TruSeq3-SE.fa:2:30:10 SLIDINGWINDOW:4:15 MINLEN:36 HEADCROP:13

Paired End) java -jar trimmomatic-0.36.jar PE -threads 36 -phred33 \${SAMPLE}_1.fastq \${SAMPLE}_2.fastq \${SAMPLE}_1.trimmed.paired.fastq \${SAMPLE}_1.trimmed.unpaired.fastq \${SAMPLE}_2.trimmed.paired.fastq \${SAMPLE}_2.trimmed.unpaired.fastq ILLUMINACLIP:TruSeq3-PE.fa:2:30:10 SLIDINGWINDOW:4:15 MINLEN:36 HEADCROP:13

Step 5: Read Mapping

Single End) hisat2 -p 36 - -dta -q -x genome -U \$SAMPLE.trim.fastq -S \$SAMPLE.sam

Paired End) hisat2 -p 36 - -dta -q -x genome -1 \${SAMPLE}_1.trimmed.paired.fastq -2 \${SAMPLE}_2.trimmed.paired.fastq -U \${SAMPLE}_1.trimmed.unpaired.fastq,\${SAMPLE}_2.trimmed.unpaired.fastq -S \$SAMPLE.sam

Step 6: File format conversion and sorting
samtools sort -@ 36 -o \$SAMPLE.bam \$SAMPLE.sam

Step 7: Merging BAM files derived from multiple lanes belonging to the same experiment

java -Xmx2g -jar picard.jar MergeSamFiles OUTPUT=\$EXPERIMENT.bam \$(for s in \$(cat samples); do echo "INPUT=\$s/\$s.bam"; done)

Step 8: **For each experiment, run StringTie using the -B and -e options in order to estimate transcript abundances from the read alignments obtained in the previous step.**

```
stringtie -eB -p 36 -G \$ANNOTATION/genes.gtf -o \$EXPERIMENT.gtf -l \$EXPERIMENT
\$EXPERIMENT.bam
```

Download the necessary scripts. A Python script (named "prepDE.py") extracts read count information directly from the files generated by StringTie.

wget http://ccb.jhu.edu/software/stringtie/dl/prepDE.py

From now on, the list of commands will be applied to each Bioproject.

Step 9: **For each Bioproject, prepare a list comprising all gtf file names and paths and name it "input.list.txt". This is a prototype of the file:**

```
\$EXPERIMENT1 <PATH\_TO\_EXPERIMENT1.gtf>\\

\$EXPERIMENT2 <PATH\_TO\_EXPERIMENT2.gtf>\\

\$EXPERIMENT3 <PATH\_TO\_EXPERIMENT3.gtf>\\

\ ...\\

\$EXPERIMENTn <PATH\_TO\_EXPERIMENTn.gtf>\\
```

Step 10: Prepare gene and transcript count matrices for each Bioproject.

```
python prepDE.py -i "input.list.txt" \
   -g "gene_count_matrix.csv" \
   -t "transcript_count_matrix.csv"
```

Additional custom scripts described within this appendix can be downloaded at the following url: http://hpc-bioinformatics.cineca.it/stress_mice/scripts/ .

Step 11: **A first custom Python script was used to prepare all meaningful configurations to test.**

```
./produce_DEGs.sh
```

Step 12: **Then, a second custom Python script is used for the three following phases. The python script invokes, in turn, other custom scripts written in R used to compute the fold change table, the list of differentially expressed genes and Gene Ontologies.**

i. produce the fold change table (once for all subsets of a bioproject):

```
python produce_lists_deseq2.py -t FC \
    -n "control" -f subsets.txt \
    -i data_dir/ -o output_dir/
```

ii. produce the DEGS tables and graphs (for each subset of a bioproject):

```
python produce_lists_deseq2.py -t DEGS \
    -n "control" -f subsets.txt \
    -i data_dir/ -o output_dir/
```

iii. produce the ontology tables and graphs (for each subset of a bioproject):

```
python produce_lists_deseq2.py -t ONTOLOGY \
    -n "control" -f subsets.txt \
    -i data_dir/ -o output_dir/ \
    -m "Mus musculus" -d "org.Mm.eg.db"
```
